# CXCR4 reduces aldosterone synthesis via regulating CYP11B2 expression

**DOI:** 10.1016/j.gendis.2025.101956

**Published:** 2025-11-28

**Authors:** Jiang Chen, Shumin Yang, Xinyue Yang, Jiayu Li, Yifan He, Chuan Peng, Wei Zhang, Yi Yang, Junlong Li, Hongji Li, Furong He, Yong Xu, Wei Huang, Jinbo Hu, Qifu Li, Linqiang Ma

**Affiliations:** a Department of Endocrinology, Sichuan-Chongqing Joint Key Laboratory of Metabolic Vascular Diseases, Chongqing Key Laboratory of Translational Medicine in Major Metabolic Diseases, The First Affiliated Hospital of Chongqing Medical University, Chongqing 400016, China; b Department of Endocrinology and Metabolism, Sichuan-Chongqing Joint Key Laboratory of Metabolic Vascular Diseases, The Affiliated Hospital of Southwest Medical University, Luzhou, Sichuan 646000, China

**Keywords:** Aldosterone, Aldosterone-producing lesions, CXCR4, CYP11B2, ID3

## Abstract

C-X-C motif chemokine receptor 4 (CXCR4) is highly expressed in aldosterone-producing adenoma, and gallium-68 pentixafor PET-CT imaging targeting CXCR4 has been utilized for subtype diagnosis in primary aldosteronism. However, the roles of CXCR4 in regulating aldosterone biosynthesis remain poorly understood. In this study, we observed a strong co-localization of aldosterone synthase (CYP11B2) and CXCR4 in aldosterone-producing adenoma and other aldosterone-producing lesions. Functional experiments in H295R cells revealed that CXCR4 overexpression significantly suppressed both aldosterone synthesis and CYP11B2 expression, whereas CXCR4 knockdown conversely enhanced aldosterone production and up-regulated CYP11B2. Mechanistically, CXCR4 inhibited aldosterone biosynthesis by up-regulating inhibitor of DNA binding (ID) proteins, which directly repressed *CYP11B2* transcription. Our data demonstrate that in aldosterone-producing lesions, CXCR4 expression is consistently elevated alongside CYP11B2, indicating a potential compensatory mechanism to counteract elevated aldosterone levels.

## Introduction

Primary aldosteronism is recognized as one of the most common causes of secondary hypertension, with a reported prevalence ranging from 4% to 22% among patients with hypertension.^1^, ^2^, ^3^ This condition arises from adrenal cortical abnormalities, such as aldosterone-producing adenoma (APA), bilateral adrenal hyperplasia, or familial hyperaldosteronism, leading to the autonomous secretion of aldosterone.

APA accounts for approximately 30%–50% of primary aldosteronism cases, and molecular investigations have primarily focused on somatic mutations in genes such as *KCNJ5*, *CACNA1D*, *ATP1A1*, *ATP2B3*, *CADM1*, and *SLC30A1*.^4^, ^5^, ^6^, ^7^, ^8^, ^9^, ^10^, ^11^ These mutations trigger calcium ion influx, which subsequently up-regulates aldosterone synthase (cytochrome P450 family 11 subfamily B member 2 or CYP11B2) at the transcriptional level.^12^, ^13^, ^14^ Although transcription factors such as ATF and CREB have been extensively studied for their roles in activating *CYP11B2* expression,^15^^,^^16^ their influence on *CYP11B1* expression suggests that additional regulatory mechanisms may exist.^17^ This underscores the need for further investigation into the transcriptional regulation of *CYP11B2*.

C-X-C chemokine receptor type 4 (CXCR4), a G protein-coupled receptor with seven transmembrane domains, is widely distributed and exhibits diverse functions.^18^ Its only known ligand, C-X-C motif chemokine ligand 12 (CXCL12), is essential for embryonic development, as evidenced by the embryonic lethality and systemic defects observed in both CXCR4-and CXCL12-knockout mice.^19^ Additionally, CXCR4 serves as a co-receptor for the human immunodeficiency virus, playing a critical role in viral entry and infection.^20^ As a chemokine receptor, CXCR4 is expressed on various hematologic malignancies and plays roles in tumor growth, proliferation, angiogenesis, and metastasis.^21^^,^^22^ Consequently, CXCR4 is regarded as a significant target for tumor therapy. The advent of gallium-68 pentixafor positron emission tomography-computed tomography (PET-CT) imaging has enabled non-invasive visualization of tissues with high CXCR4 expression, showing promising diagnostic potential in oncology.^23^ It has been found that CXCR4 is highly expressed in aldosterone-producing tissue, coinciding with the expression pattern of aldosterone synthase,^24^ but the specific molecular mechanisms remain unclear. Gallium-68 pentixafor PET-CT imaging targeting CXCR4 is considered to be of high value in the diagnosis and classification of primary aldosteronism.^25^, ^26^, ^27^, ^28^ With a diagnostic concordance rate with adrenal vein sampling as high as 90%, especially in patients with a typical unilateral adrenal nodule greater than 10 mm, this imaging technique offers a non-invasive diagnostic option for these patients.^29^, ^30^, ^31^, ^32^ However, the role of CXCR4 in aldosterone regulation and the mechanisms underlying its up-regulation in APA have yet to be fully elucidated.

The present study aims to investigate the role of CXCR4 in aldosterone biosynthesis and its potential involvement in the pathophysiology of primary aldosteronism. By exploring the regulatory effects of CXCR4 on steroidogenic pathways, this study seeks to provide new insights into its clinical and biological significance.

## Materials and methods

### Subjects

Adrenal tissue samples were collected from patients diagnosed with non-functional adenoma (NFA) and unilateral primary aldosteronism (UPA) who underwent laparoscopic adrenalectomy in the Department of Endocrinology at the First Affiliated Hospital of Chongqing Medical University. Diagnosis of NFA and UPA followed established criteria.^30^^,^^33^ Sanger sequencing was performed on adrenal samples from UPA patients with morphologically visible nodules, targeting high-frequency mutation sites in the *KCNJ5*, *ATP1A1*, and *ATP2B3* genes. PCR amplification was carried out using primers listed in Table S1. For UPA samples lacking mutations in these genes, whole-exome sequencing (Novogene Co., Ltd., China) was conducted on CYP11B2-positive regions. This study was approved by the Ethics Committee of the First Affiliated Hospital of Chongqing Medical University (Approval No. 2024-322-01). Written informed consent was obtained from all participants before their inclusion in the study.

### Immunohistochemical staining and quantification

Immunohistochemical staining was performed on adrenal tissue samples from 20 NFA patients and 56 UPA patients. The primary antibodies used were rabbit anti-CXCR4 (1:500, Abcam, ab124824) and mouse anti-CYP11B2 (1:100, Millipore, mabs1251). The detailed protocol has been previously described.^34^ All stained sections were scanned and captured by Olympus VS200 (Olympus, Japan). Quantification of immunohistochemistry images was conducted using FIJI software. For each tissue section, three randomly selected fields from the target region were analyzed, and quantification was based on the average integrated optical density. To ensure consistency across samples, all staining procedures were standardized and conducted within six months. The specificity of the antibodies was confirmed using negative controls and isotype IgG controls (Fig. S1).

### Immunofluorescence staining

Immunofluorescence staining was performed on adrenal samples from 1 NFA patient and 12 UPA patients. The primary antibodies used were rabbit anti-CXCR4 (1:100, Abcam, ab124824) and mouse anti-CYP11B2 (1:100, Millipore, mabs1251). The corresponding secondary antibodies were goat anti-rabbit Alexa Fluor™ Plus 488 (Thermo Fisher Scientific) and goat anti-mouse Alexa Fluor™ Plus 555 (Thermo Fisher Scientific), respectively. The detailed protocol has been previously described.^34^ All stained sections were scanned and captured by Olympus VS200 (Olympus, Japan).

### RNA-sequencing data analysis

Total RNA was extracted from cells using Trizol reagent (Roche, 11667165001). Subsequently, quality assessment, library construction, library quality control, and sequencing were performed by Shanghai Applied Protein Technology Co., Ltd., Shanghai, China. High-quality RNA samples were enriched using magnetic beads coated with Oligo (dT). mRNA was fragmented and reverse-transcribed to generate first-strand cDNA, followed by second-strand synthesis. Double-stranded cDNA was purified, end-repaired, A-tailed, and ligated to sequencing adapters. After size selection with AMPure XP beads, the cDNA library was quantified and sequenced. Bioinformatics analysis included raw sequencing data analysis, sequencing data quality assessment, alignment to the reference genome, gene expression quantification, differential gene expression analysis, and functional enrichment analysis.

### Cell culture and reagents

The human adrenocortical carcinoma cell line (H295R) was purchased from Procell (Wuhan, China). Cells were maintained in DMEM/F12 (Gibco) supplemented with 10% fetal bovine serum (Gibco) and 1% insulin-transferrin-selenium (Gibco) at 37 °C in an atmosphere of 5% CO_2_.

### Lentiviral infection

Lentiviruses (LV-CXCR4, LV-ID3-3XFLAG, LV-shCXCR4, LV-shID3) were purchased from Obio Technology (Shanghai, China). H295R cells were infected with the lentivirus when they reached 50% confluence. After a 16-h incubation, the medium was replaced. Seventy-two hours post-infection, stable cell lines were selected using puromycin (Gibco, A1113803) or blasticidin S (Beyotime, ST018-5 ml), achieving > 95% transduction efficiency. The shRNA sequences used were: LV-shCtrl, CCTAAGGTTAAGTCGCCCTCG; LV-shCXCR4, TCCTGTCCTGCTATTGCATTA; LV-shID3, TCAGCTTAGCCAGGTGGAAAT. The overexpression and knockdown efficiencies were detected using Western blotting and quantitative PCR.

### Assay of aldosterone and related hormones

The supernatant of H295R cells was collected, and the hormone concentrations were measured using UltriMate Thermo 3000 UPLC and TSQ Endura triple quadrupole mass spectrometer (Thermo Fisher Scientific). Steroid hormone production was normalized to total cellular protein levels. All steroid hormone assays were performed in three independent biological replicates.

### Western blotting

Cells were lysed using RIPA buffer containing protease and phosphatase inhibitors (Thermo Fisher Scientific). The cell lysates were centrifuged to retain supernatant, and the protein concentration was determined using a BCA protein assay kit (Beyotime, P0009). Equal amounts of protein were separated by 10% or 15% Tris-Glycine PAGE (Beyotime, P0455M) and transferred onto a PVDF membrane at 250 mA for 90 min. Membranes were blocked with 5% non-fat dry milk in Tris-buffered saline-Tween 20 at room temperature on a shaker for 1 h. Primary antibodies were applied at a dilution of 1:1000 and incubated at 4 °C overnight. After washing, membranes were incubated with secondary antibodies at a dilution of 1:5000 on a shaker at room temperature for 1 h. Protein bands were visualized using the WesternBright ECL kit (Advansta, K-12045-D50). The primary antibodies included anti-CYP11B2-mouse (Millipore, mabs1251), anti-CXCR4-rabbit (Abcam, ab124824), anti-GAPDH-rabbit (Servicebio, GB11002), anti-FLAG tag-rabbit (Cell Signaling Technology, 14793S), anti-ID3-rabbit (Cell Signaling Technology, 9837), and anti-ID1-mouse (Santa Cruz Biotechnology, sc-133104), and the secondary antibodies included goat-anti-rabbit HRP and goat-anti-mouse HRP (Invitrogen). For Western blotting analysis, protein signals were detected using a CCD imaging system, and exposure times were adjusted according to signal intensity.

### RNA isolation and quantitative PCR

Total cellular RNA was extracted using Trizol reagent (Roche, 11667165001). Genomic DNA was removed, and reverse transcription was performed using the PrimeScript™ FAST RT reagent Kit with gDNA Eraser (Takara, RR092A) to generate cDNA. Quantitative reverse transcription PCR was carried out using TB Green® Premix Ex Taq™ II (Takara, RR820A) according to the manufacturer's protocol. Each quantitative reaction was performed in triplicate. Human GAPDH was used as an internal reference to normalize the data presentation. The primer sequences are provided in Table S2.

### Statistical analysis

All statistical analyses were performed using GraphPad Prism 9 software. Data were presented as mean ± standard deviation unless otherwise stated. For normally distributed data, comparisons between two groups were made using a two-sample Student's *t*-test. Multiple group comparisons were analyzed using one-way ANOVA followed by Tukey's post hoc test for pairwise comparisons. A *P*-value < 0.05 was considered statistically significant. Data visualization and statistical result presentations were conducted using GraphPad Prism 9 software. The histological images presented were representative of biological replicates.

## Results

### CXCR4 expression in aldosterone-producing lesions is significantly higher than in NFA and highly correlated with CYP11B2 expression

**Figure 1:**
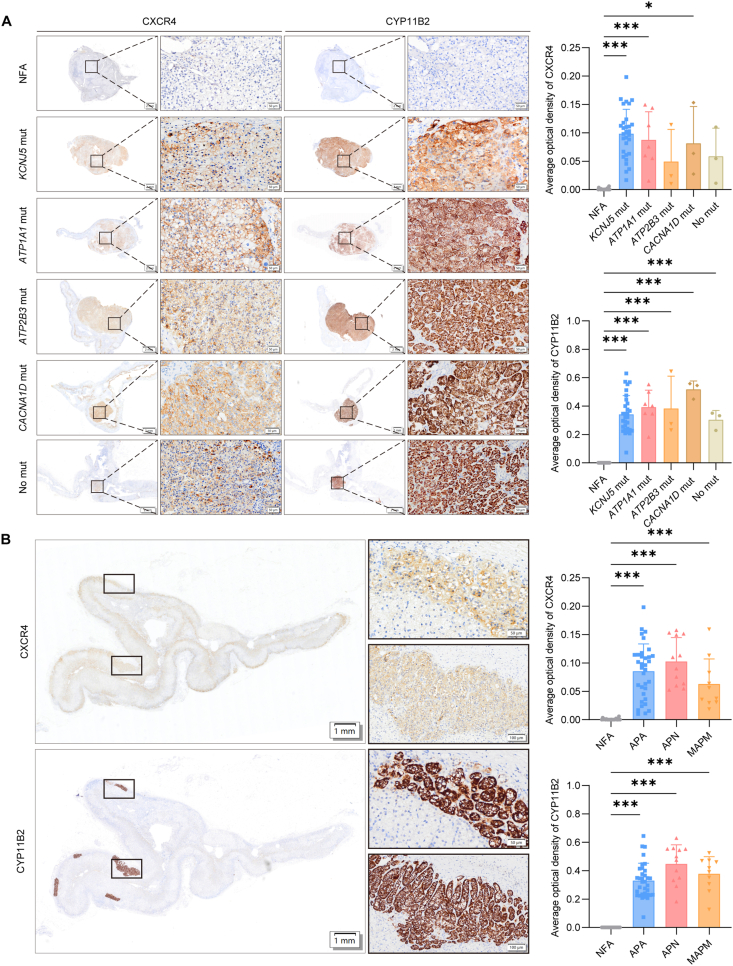
CXCR4 is highly expressed in aldosterone-producing lesions. **(A)** Immunohistochemical staining and corresponding average optical density values of CXCR4 and CYP11B2 in the adrenal samples of NFA (*n* = 20), *KCNJ5*-mutated UPA (*n* = 30), *ATP1A1*-mutated UPA (*n* = 7), *ATP2B3*-mutated UPA (*n* = 3), *CACNA1D*-mutated UPA (*n* = 3), and UPA with no mutations (*n* = 3). Scale bars, 2 mm or 50 μm. **(B)** Immunohistochemical staining of CXCR4 and CYP11B2 in MAPM and average optical density values of CXCR4 and CYP11B2 in the adrenal samples of NFA (*n* = 20), APA (*n* = 34), APN (*n* = 12), and MAPM (*n* = 10). Scale bars, 1 mm, 100 μm, 50 μm. The data were presented as mean ± standard deviation. ∗*P* < 0.05, ∗∗*P* < 0.01, and ∗∗∗*P* < 0.001. NFA, non-functional adenoma; UPA, unilateral primary aldosteronism; APA, aldosterone-producing adenoma; APN, aldosterone-producing nodule; MAPM, multiple aldosterone-producing micronodule.

Previous studies^24^ have reported high expression of CXCR4 in aldosterone-producing tissues, including APA. To confirm these findings, we compared CXCR4 expression in adrenal samples from 20 patients with NFA and 56 patients with UPA using immunohistochemical staining. Of the 56 UPA cases, 46 adrenal samples with prominent nodular morphology underwent gene sequencing. The identified mutations included KCNJ5 (30 cases), ATP1A1 (7 cases), ATP2B3 (3 cases), and CACNA1D (3 cases), with no mutation found in 3 cases. Our data show that CXCR4 expression was significantly higher in mutation-driven aldosterone-producing lesions compared with NFA (Fig. 1A). However, there were no significant differences in CXCR4 expression across different gene mutation types (Fig. 1A). Given the diagnostic value of gallium-68 pentixafor PET-CT imaging for lesions larger than 10 mm, we next explored the relationship between CXCR4 expression and lesion size. Based on pathological diagnoses,^35^ the 56 UPA cases were categorized into 34 APA cases, 12 aldosterone-producing nodule cases, and 10 multiple aldosterone-producing micronodule cases. In accordance with previous studies,^9^^,^^36^^,^^37^ aldosterone-producing lesions with *ATP1A1*, *ATP2B3*, and *CACNA1D* mutations were typically smaller than those with *KCNJ5* mutations, often presenting as aldosterone-producing nodules. Nevertheless, CXCR4 expression was significantly elevated in APA, aldosterone-producing nodules, and multiple aldosterone-producing micronodules compared with NFA (Fig. 1A and B), suggesting that CXCR4 expression is independent of lesion size.

To further delineate the expression patterns of CXCR4 and CYP11B2, immunofluorescence labeling of both markers was performed in NFA and UPA adrenal samples. Our findings revealed that in aldosterone-producing lesions, both CXCR4 and CYP11B2 were highly expressed and exhibited consistent distribution, with CXCR4 localized to the cell membrane and CYP11B2 localized to the cytoplasm (Fig. S2). These findings suggest a potential correlation between the expression of CXCR4 and CYP11B2 in aldosterone-producing lesions.

### Overexpression of CXCR4 inhibits aldosterone synthesis

**Figure 2:**
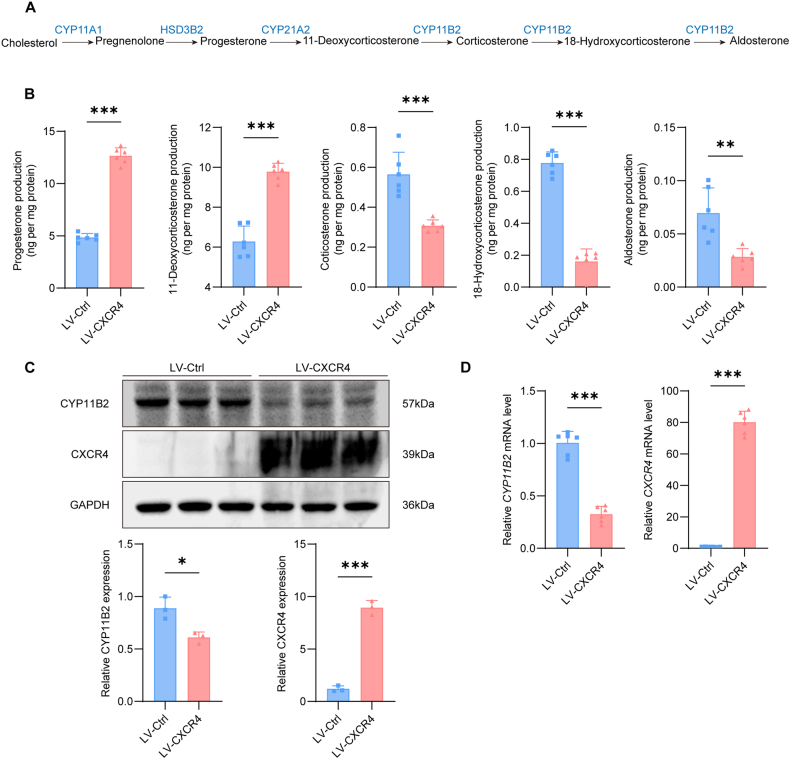
Overexpression of CXCR4 decreases aldosterone synthesis in H295R cells. **(A)** Schematic representation of the aldosterone synthesis pathway in H295R cells. **(B)** Steroid hormone levels (ng per mg protein) in the supernatant of H295R cells after CXCR4 overexpression, including progesterone, 11-deoxycorticosterone, corticosterone, 18-hydroxycorticosterone, and aldosterone (*n* = 6). **(C)** Western blotting analysis and quantification of indicated proteins in H295R cells with CXCR4 overexpression (*n* = 3). **(D)***CYP11B2* and *CXCR4* mRNA expression in H295R cells with CXCR4 overexpression (*n* = 6). The data were presented as mean ± standard deviation. ∗*P* < 0.05, ∗∗*P* < 0.01, and ∗∗∗*P* < 0.001.

Given the highly consistent expression of CXCR4 with CYP11B2, we asked whether CXCR4 regulates aldosterone synthesis. We established a stable H295R cell line overexpressing CXCR4 (LV-CXCR4). The synthesis of aldosterone and related hormones, as well as the expression of aldosterone synthase CYP11B2, were assessed. The key steps in aldosterone synthesis pathway were reviewed (Fig. 2A). Mass spectrometry revealed that CXCR4 overexpression significantly reduced the synthesis of corticosterone, 18-hydroxycorticosterone, and aldosterone in H295R cells, while upstream substrates such as progesterone and 11-deoxycorticosterone exhibited accumulative increases (Fig. 2B). Since the final steps of aldosterone synthesis are catalyzed by CYP11B2, where deoxycorticosterone undergoes 11β- and 18-hydroxylation followed by 18-oxidation, a significant reduction in aldosterone and its key precursors suggests that CXCR4 may regulate aldosterone biosynthesis through modulation of CYP11B2 expression. To test this hypothesis, the expression levels of CYP11B2 were assessed. Consistent with the observed hormone changes, Western blotting and real-time quantitative PCR confirmed significant reductions in both protein and mRNA levels of CYP11B2 (Fig. 2C and D).

Additionally, the synthesis of various steroid hormones in H295R cells was assessed following the overexpression of CXCR4 (Fig. S3A–S3E). A significant reduction in cortisol levels was observed (Fig. S3C), along with decreased synthesis of 18-hydroxycortisol and 18-oxocortisol, both catalyzed by CYP11B2 (Fig. S3D and S3E). Similarly, there was an accumulation of upstream substrates, 17α-hydroxypregnenolone and 17α-hydroxyprogesterone, in the cortisol biosynthesis pathway (Fig. S3A and S3B). As CYP11B2 demonstrates limited 11β–hydroxylase activity *in vitro*,^38^ mRNA expression levels of all steroidogenic enzymes were analyzed to elucidate the mechanism underlying the reduction in cortisol synthesis. Minor reductions were noted for *STAR*, *CYP11A1*, *HSD3B2*, *CYP21A2*, and *CYP17A1* (Fig. S3F–S3J), while *CYP11B1* was decreased by nearly 50% (Fig. S3K), correlating with the observed cortisol decline. Together, these findings indicate a disruption in overall steroid hormone synthesis in H295R cells in response to CXCR4 overexpression. In summary, these results suggest that CXCR4 negatively regulates aldosterone synthesis *in vitro*.

### Knockdown of CXCR4 promotes aldosterone synthesis

**Figure 3:**
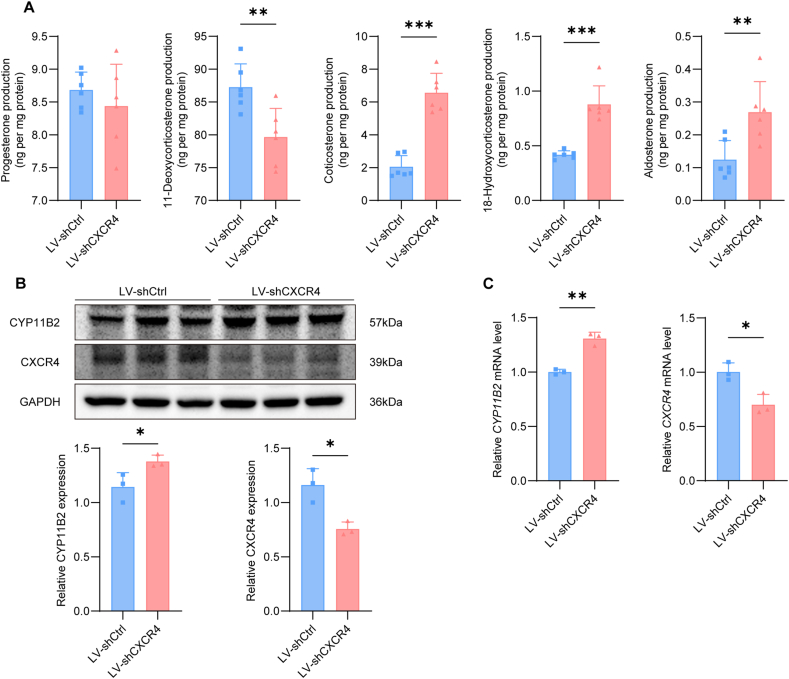
Knockdown of CXCR4 promotes aldosterone synthesis in H295R cells. **(A)** Steroid hormone levels (ng per mg protein) in the supernatant of H295R cells after CXCR4 knockdown, including progesterone, 11-deoxycorticosterone, corticosterone, 18-hydroxycorticosterone, and aldosterone (*n* = 6). **(B)** Western blotting analysis and quantification of indicated proteins in H295R cells with CXCR4 knockdown (*n* = 3). **(C)***CYP11B2* and *CXCR4* mRNA expression in H295R cells with CXCR4 knockdown (*n* = 3). The data were presented as mean ± standard deviation. ∗*P* < 0.05, ∗∗*P* < 0.01, and ∗∗∗*P* < 0.001.

To further validate the regulatory effect of CXCR4 on aldosterone synthesis, we constructed a stable cell line with CXCR4 knockdown (LV-shCXCR4) and similarly assessed the synthesis of steroid hormones and the expression changes of CYP11B2. CXCR4 knockdown led to a significant increase in the synthesis of corticosterone, 18-hydroxycorticosterone, and aldosterone, while the level of upstream substrate 11-deoxycorticosterone decreased markedly (Fig. 3A). Additionally, both protein and mRNA levels of CYP11B2 were significantly up-regulated following CXCR4 knockdown (Fig. 3B and C). Further analysis of the synthesis of other steroid hormones revealed a significant up-regulation of cortisol, along with 18-hydroxycortisol and 18-oxocortisol (Fig. S4A–S4E). Real-time quantitative PCR was subsequently performed to examine the mRNA expression levels of other steroidogenic enzymes (Fig. S4F–S4K), revealing a significant increase in *CYP11B1* mRNA levels (Fig. S4K). Collectively, these findings indicate that CXCR4 negatively regulates CYP11B2 expression and aldosterone synthesis.

### RNA sequencing reveals down-regulation of the aldosterone synthesis pathway

**Figure 4:**
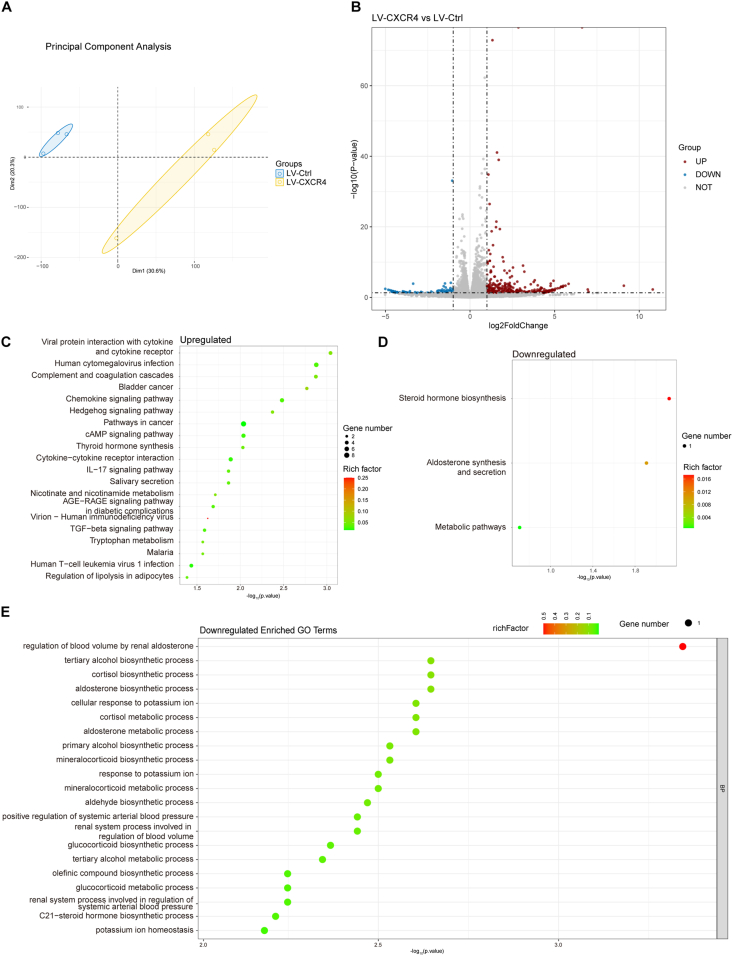
RNA sequencing reveals down-regulation of the aldosterone synthesis pathway. **(A, B)** Principal component analysis (A) and volcano plot (B) of transcriptomic analysis of H295R cells after CXCR4 overexpression. **(C)** Up-regulated pathways indicated by Kyoto Encyclopedia of Genes and Genomes (KEGG) enrichment analysis. **(D)** Down-regulated pathways indicated by KEGG enrichment analysis. **(E)** Down-regulated biological processes indicated by Gene Ontology (GO) functional enrichment analysis.

To elucidate the specific mechanisms by which CXCR4 regulates aldosterone synthesis, RNA sequencing was performed on H295R cells overexpressing CXCR4. Principal component analysis revealed significant transcriptional changes, identifying 460 differentially expressed genes, with 364 up-regulated and 96 down-regulated (Fig. 4A and B). Kyoto Encyclopedia of Genes and Genomes (KEGG) enrichment analysis indicated that pathways such as viral protein interaction with cytokine and cytokine receptor, human cytomegalovirus infection, and complement and coagulation cascades were significantly up-regulated in the LV-CXCR4 cell line, consistent with CXCR4's role as a chemokine receptor (Fig. 4C). In contrast, pathways involved in steroid hormone biosynthesis, aldosterone synthesis and secretion, and metabolic pathways were significantly down-regulated (Fig. 4D). Additionally, Gene Ontology (GO) functional enrichment also confirmed significant down-regulation of aldosterone biosynthesis and metabolic processes (Fig. 4E).

### CXCR4 inhibits aldosterone synthesis via up-regulation of ID3

**Figure 5:**
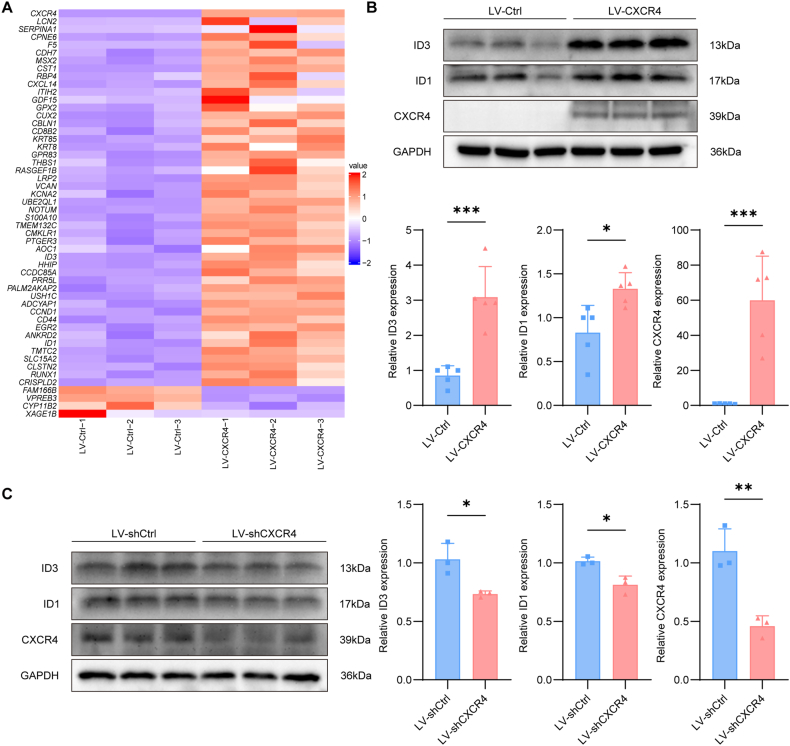
CXCR4 regulates the expression of ID3 and ID1. **(A)** Heatmap of differentially expressed genes in H295R cells after CXCR4 overexpression. **(B, C)** Western blotting analysis and quantification of indicated proteins in H295R cells with CXCR4 overexpression **(B)** (*n* = 5) or knockdown **(C)** (*n* = 3). The data were presented as mean ± standard deviation. ∗*P* < 0.05, ∗∗*P* < 0.01, and ∗∗∗*P* < 0.001.

**Figure 6:**
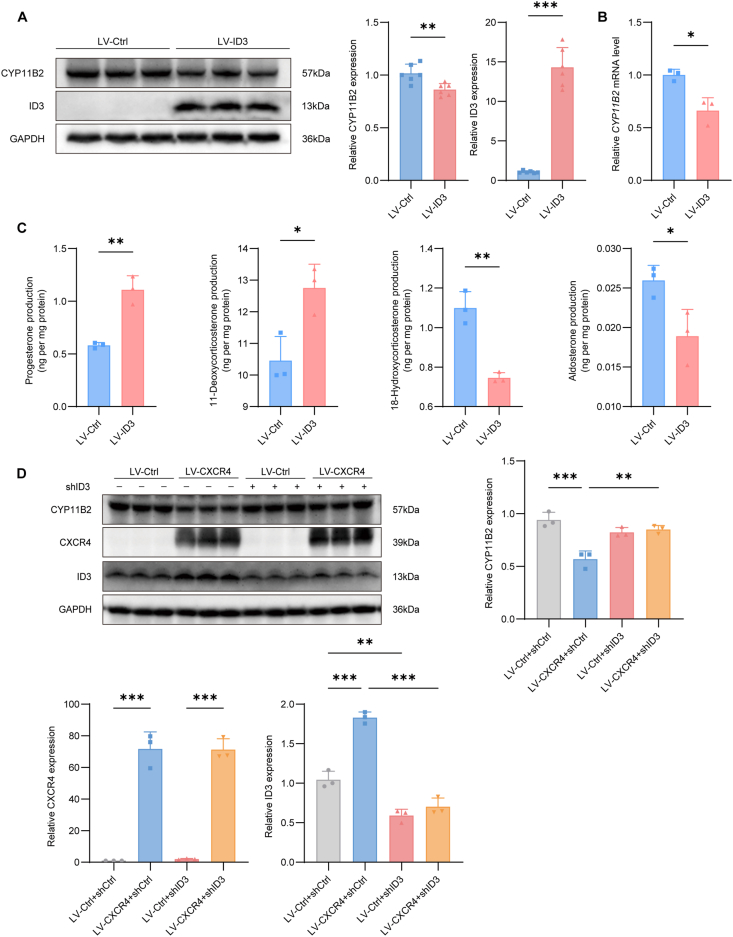
CXCR4 inhibits aldosterone synthesis via up-regulation of ID3. **(A)** Western blotting analysis and quantification of indicated proteins in H295R cells with ID3 overexpression (*n* = 6). **(B)***CYP11B2* mRNA expression in H295R cells with ID3 overexpression (*n* = 3). **(C)** Steroid hormone levels (ng per mg protein) in the supernatant of H295R cells after ID3 overexpression, including progesterone, 11-deoxycorticosterone, 18-hydroxycorticosterone, and aldosterone (*n* = 3). **(D)** Western blotting analysis and quantification of indicated proteins in H295R cells overexpressing CXCR4 with or without ID3 knockdown (*n* = 3). The data were presented as mean ± standard deviation. ∗*P* < 0.05, ∗∗*P* < 0.01, and ∗∗∗*P* < 0.001.

The RNA sequencing data revealed that CXCR4 overexpression in H295R cells led to a significant reduction in *CYP11B2* transcription (Fig. 5A), suggesting that this may be the primary reason for the observed reduction in aldosterone synthesis. Notably, the transcriptional repressors ID1 and ID3 were among the most up-regulated genes (Fig. 5A). Validation experiments confirmed that CXCR4 overexpression in H295R cells up-regulated ID3 and ID1 expression (Fig. 5B), while CXCR4 knockdown resulted in significant down-regulation of both (Fig. 5C). We hypothesized that ID proteins mediate CXCR4's repressive effect on CYP11B2. Given that ID3 exhibited greater expression and more pronounced changes in LV-CXCR4 cells, we constructed a stable H295R cell line overexpressing ID3 (LV-ID3-3XFLAG). The protein and mRNA levels of CYP11B2 were significantly decreased following the overexpression of ID3 (Fig. 6A and B). Concurrently, the synthesis of 18-hydroxycorticosterone and aldosterone, catalyzed by CYP11B2, was significantly reduced (Fig. 6C). Additionally, the concentrations of upstream substrates such as progesterone and 11-deoxycorticosterone accumulated (Fig. 6C). To assess the potential transcriptional regulatory effects of ID3 on other steroidogenic enzymes, mRNA expression levels were analyzed (Fig. S5A–S5F). The results indicated minimal changes in the mRNA levels of most enzymes, while *CYP11B1* mRNA levels showed a significant decrease (Fig. S5F). Mass spectrometry analysis of steroid hormones involved in cortisol synthesis showed that cortisol, along with the CYP11B2-catalyzed metabolites 18-hydroxycortisol and 18-oxocortisol, was significantly reduced (Fig. S5G–S5K).

Given the marked up-regulation of ID3 following CXCR4 overexpression, we next assessed whether silencing ID3 could rescue CYP11B2 expression. In H295R cells stably overexpressing CXCR4, we generated additional cell lines transduced with either a control lentivirus or an ID3-targeting shRNA lentivirus. Our results demonstrated that knockdown of ID3 effectively restored CYP11B2 expression suppressed by CXCR4 overexpression (Fig. 6D). These results indicate that CXCR4 reduces aldosterone synthesis through ID3-mediated regulation of *CYP11B2* transcription.

## Discussion

The pathogenesis of APA has predominantly focused on dysregulated calcium ion homeostasis,^33^ with less attention given to the regulation of CYP11B2 expression itself. In this study, we observed that in H295R cells, CXCR4 up-regulates the transcriptional repressor ID3, leading to a reduction in *CYP11B2* transcription and inhibition of aldosterone synthesis. These results implicate CXCR4 as a novel regulator of aldosterone production in pathological lesions.

Noninvasive subtyping of primary aldosteronism remains clinically essential. Gallium-68 pentixafor PET-CT, which targets CXCR4 overexpression, has emerged as a promising diagnostic tool.^27^^,^^30^^,^^32^ In our study, despite inter-lesional heterogeneity in CXCR4 expression across aldosterone-producing lesions, its levels remained uncorrelated with lesion size or genetic mutation status. These findings provide mechanistic insights and theoretical support for the clinical application of Gallium-68 pentixafor PET-CT in primary aldosteronism subtype diagnosis.

CXCR4 overexpression is widely recognized in various malignancies and has traditionally been associated with tumorigenesis and disease progression.^39^ For instance, CXCR4 signaling has been implicated as a central component of the immunosuppressive tumor microenvironment in hepatocellular carcinoma.^40^ However, recent evidence suggests that strategies aimed at promoting CXCR4 clustering on the tumor cell surface may paradoxically suppress metastasis.^41^ Motivated by these findings, we sought to investigate the functional role of CXCR4 in aldosterone biosynthesis. Unexpectedly, our results revealed that CXCR4 markedly down-regulated CYP11B2 expression and suppressed aldosterone production in H295R cells. These findings uncover a previously unrecognized role for CXCR4 in the regulation of steroid hormone synthesis, particularly aldosterone, and suggest novel therapeutic implications for managing primary aldosteronism.

The diagnosis and management of Cushing's syndrome remain challenging. Among the ACTH-independent etiologies, the most common one is unilateral adrenal adenomas, also known as cortisol-producing adenomas.^42^ Previous studies^24^ have reported elevated CXCR4 expression in cortisol-producing adenomas, and CXCR4-targeted Gallium-68 pentixafor PET-CT imaging has shown potential for diagnosing Cushing's syndrome.^43^ In our experiment, CXCR4 was also found to significantly reduce the expression of CYP11B1 and the synthesis of cortisol, suggesting that CXCR4 may similarly attenuate hypercortisolemia in cortisol-producing adenomas.

The ID protein family, acting as transcriptional repressors, plays a significant role in the development and progression of cancer.^44^, ^45^, ^46^ ID proteins bind to basic helix-loop-helix (bHLH) transcription factors, forming heterodimers that lack DNA-binding domains, thereby preventing these factors from interacting with DNA.^47^^,^^48^ Down-regulation of ID3 in B cells allows E proteins to activate key genes such as CXCR4 to regulate B cell differentiation.^49^ Our data indicated that CXCR4 directly influenced ID3 expression, thereby expanding the known transcriptional regulatory network associated with ID3. While ID proteins are generally regarded as potential proto-oncogenes, their anti-tumor roles have become increasingly recognized. ID3 has been reported to exert potent anti-tumor activity in liver Kupfer cells by binding to the transcription factors ELK1 and E2A.^50^ In our study, overexpression of ID3 significantly reduced *CYP11B2* transcription and aldosterone synthesis, underscoring the crucial role of ID3 in the transcriptional regulation of *CYP11B2* and its potential implications for primary aldosteronism treatment. Furthermore, the regulatory effect of ID3 on the transcriptional level of *CYP11B1* may be attributed to the conserved transcriptional regulatory elements between *CYP11B1* and *CYP11B2*.^15^^,^^17^

Nevertheless, this study has several limitations. Some recently reported rare mutations (*e.g.*, *CADM1*, *SLC30A1*) were not represented in our cohort. Additionally, sequencing of micronodules was limited by technical constraints. Although H295R cells are widely used for studying adrenal steroidogenesis, they lack the zonal architecture of the adrenal cortex and may not fully reflect the physiological environment. Furthermore, the lack of an APA animal model restricts functional validation. While our data support a central role for ID3 in *CYP11B2* regulation, the detailed mechanisms remain to be elucidated. Given the overlapping functions between ID3 and ID1, the potential contribution of ID1 to CYP11B2 regulation cannot be excluded.

In summary, our findings indicate that CXCR4 modulates CYP11B2 expression and aldosterone synthesis through ID3. This finding leads us to hypothesize that the increased expression of CXCR4 in aldosterone-producing lesions may function as a compensatory mechanism to reduce high aldosterone levels. Additionally, our study provides a molecular basis for the clinical application of Gallium-68 pentixafor PET-CT imaging technology in the diagnosis and classification of primary aldosteronism.

## CRediT authorship contribution statement

**Jiang Chen:** Writing – review & editing, Writing – original draft, Validation, Methodology, Data curation, Conceptualization. **Shumin Yang:** Writing – review & editing, Resources, Project administration, Funding acquisition, Data curation, Conceptualization. **Xinyue Yang:** Resources, Methodology, Investigation, Data curation, Conceptualization. **Jiayu Li:** Resources, Methodology, Investigation, Data curation, Conceptualization. **Yifan He:** Methodology, Investigation, Formal analysis. **Chuan Peng:** Writing – review & editing, Supervision. **Wei Zhang:** Resources. **Yi Yang:** Resources, Funding acquisition. **Junlong Li:** Resources. **Hongji Li:** Validation, Data curation. **Furong He:** Data curation. **Yong Xu:** Resources. **Wei Huang:** Resources. **Jinbo Hu:** Writing – review & editing, Supervision, Project administration, Funding acquisition, Data curation. **Qifu Li:** Writing – review & editing, Supervision, Resources, Project administration, Funding acquisition, Data curation. **Linqiang Ma:** Writing – review & editing, Writing – original draft, Supervision, Project administration, Methodology, Funding acquisition, Data curation.

## Funding

This work is supported by the 10.13039/501100001809National Natural Science Foundation of China (No. U21A20355, 82170825, 82100833), the National key research & development plan of China, major project of prevention and treatment for common diseases (No. 2022YFC2505300; sub-project: No. 2022YFC2505301, 2022YFC2505302, 2022YFC2505306), the Joint Medical Research Project of Chongqing Science and Technology Commission & Chongqing Health and Family Planning Commission (Major Project) (China) (No. 2022ZDXM003), the Science and Technology Research Project for Young Scholars of Chongqing Education Commission (China) (No. KJQN202300406), and the Chongqing Medical Scientific Research Project (Joint Project of Chongqing Health Commission and Science and Technology Bureau) (No. 2025GGXM004).

## Conflict of interests

The author declared no conflict of interests.
