# Supplementary material for: CXCR4 reduces aldosterone synthesis via regulating CYP11B2 expression
Source: Genes Dis. 2025 Nov 28;13(5):101956. doi: 10.1016/j.gendis.2025.101956 (PMC13264065; doi:10.1016/j.gendis.2025.101956)
Supplement: Multimedia component 1 [file mmc1.docx]

**DATA SUPPLEMENT**

**CXCR4 Reduces Aldosterone Synthesis via Regulating CYP11B2 Expression**

Jiang Chen ^a^, Shumin Yang ^a^, Xinyue Yang ^a^, Jiayu Li ^a^, Yifan He ^a^, Chuan Peng ^a^, Wei Zhang ^a^, Yi Yang ^a^, Junlong Li ^a^, Hongji Li ^a^, Furong He ^a^, Yong Xu ^b^, Wei Huang ^b^, Jinbo Hu ^a^, Qifu Li ^a^, Linqiang Ma ^a^

^a^ Department of Endocrinology, Sichuan-Chongqing Joint Key Laboratory of Metabolic Vascular Diseases, Chongqing Key Laboratory of Translational Medicine in Major Metabolic Diseases, the First Affiliated Hospital of Chongqing Medical University, Chongqing 400016, China.

^b^ Department of Endocrinology and Metabolism, Sichuan-Chongqing Joint Key Laboratory of Metabolic Vascular Diseases, the Affiliated Hospital of Southwest Medical University, Luzhou, Sichuan 646000, China

**Co-first authors:** Jiang Chen, Shumin Yang, Xinyue Yang and Jiayu Li contributed equally as cofirst authors.

**Correspondence to:**

Linqiang Ma, the First Affiliated Hospital of Chongqing Medical University, No. 1 Youyi Road, Yuzhong District, Chongqing 400016, China, Email [tom_linqiang@163.com](mailto:tom_linqiang@163.com);

Qifu Li, the First Affiliated Hospital of Chongqing Medical University, No. 1 Youyi Road, Yuzhong District, Chongqing 400016, China, Email [liqifu@yeah.net](mailto:liqifu@yeah.net);

Jinbo Hu, the First Affiliated Hospital of Chongqing Medical University, No. 1 Youyi Road, Yuzhong District, Chongqing 400016, China, Email [hujinbo@cqmu.edu.cn](mailto:hujinbo@cqmu.edu.cn)

**Supplemental materials:** Tables S1-S2 and Figures S1-S9

**Supplemental materials**

**Table S1. Sequences of primers used for Sanger Sequencing**

|  | | | |
| --- | --- | --- | --- |
| **Primer target** | **Forward primer (5' to 3')** | **Reverse primer (5' to 3')** | **Amplified fragment size（bp）** |
| *KCNJ5* | AACCAGGACATGGAGATTGG | CCACGTTGATGTCTGTCTGG | 751bp |
| *ATP1A1* | CCACTACTCCTGAATGGATC | TCCTCTTCTGTAGCAGCTTG | 121bp |
| *ATP2B3* | GATTGAGACGTTTGTCGTGG | CCTTGACAGAGTAAGCTAAGG | 167bp |

**Table S2. Sequences of primers utilized in RT-qPCR.**

|  | | | |
| --- | --- | --- | --- |
| **Primer target** | **Forward primer (5' to 3')** | **Reverse primer (5' to 3')** | **Amplified fragment size（bp）** |
| *CXCR4* | ACTACACCGAGGAAATGGGCT | CCCACAATGCCAGTTAAGAAGA | 133bp |
| *GAPDH* | GGAGCGAGATCCCTCCAAAAT | GGCTGTTGTCATACTTCTCATGG | 197bp |
| *CYP11B2* | CCCTCAACACTACACAGGCA | GTCATCAGCAAGGGAAACGC | 132bp |
| *CYP11B1* | AGTGCTGCCCTTTGAAGCCAT | CTTCCAGGTGCAGGTCCTCAT | 101bp |
| *ID3* | GAGAGGCACTCAGCTTAGCC | TCCTTTTGTCGTTGGAGATGAC | 170bp |
| *ID1* | ACGACATGAACGGCTGTTACTCAC | GAATCTCCACCTTGCTCACCTTGC | 84bp |
| *STAR* | GGGAGTGGAACCCCAATGTC | CCAGCTCGTGAGTAATGAATGT | 78bp |
| *CYP11A1* | GCAGTGTCTCGGGACTTCG | GGCAAAGCGGAACAGGTCA | 102bp |
| *HSD3B2* | CTTGTGCGTTAAGACCCACAT | GGGTTGACTGTAGAGAACTTTCC | 124bp |
| *CYP21A2* | CAAGCTGGTGTCTAGGAACTACC | TCTCATGCGCTCACAGAACTC | 163bp |
| *CYP17A1* | GCTGCTTACCCTAGCTTATTTGT | ACCGAATAGATGGGGCCATATTT | 174bp |

**
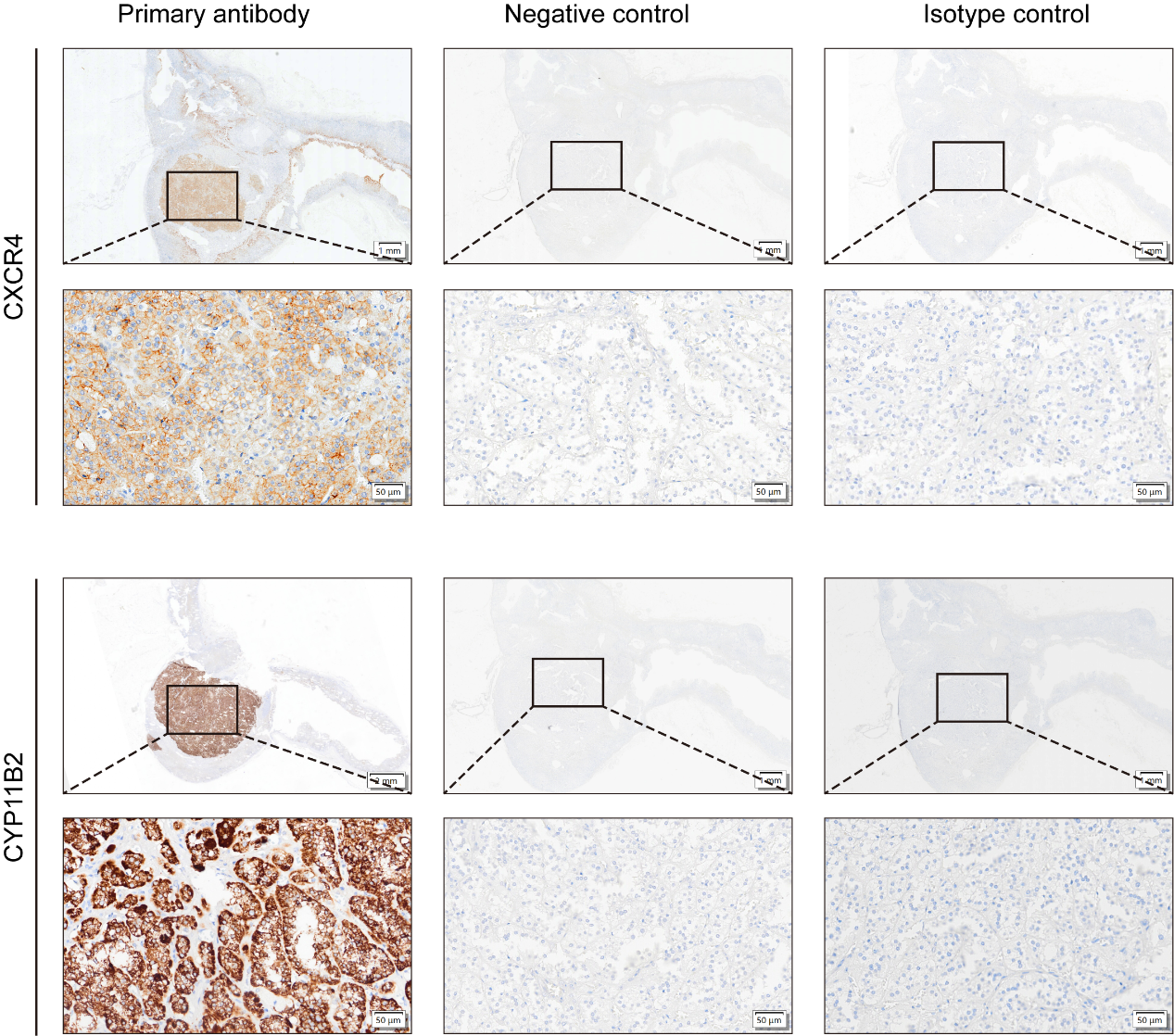
**

**Figure S1.** **Immunohistochemical staining of CXCR4 and CYP11B2 in UPA tissues using specific primary antibodies, along with corresponding negative and isotype IgG controls. Scale bars, 1 mm, 50 μm.**

**
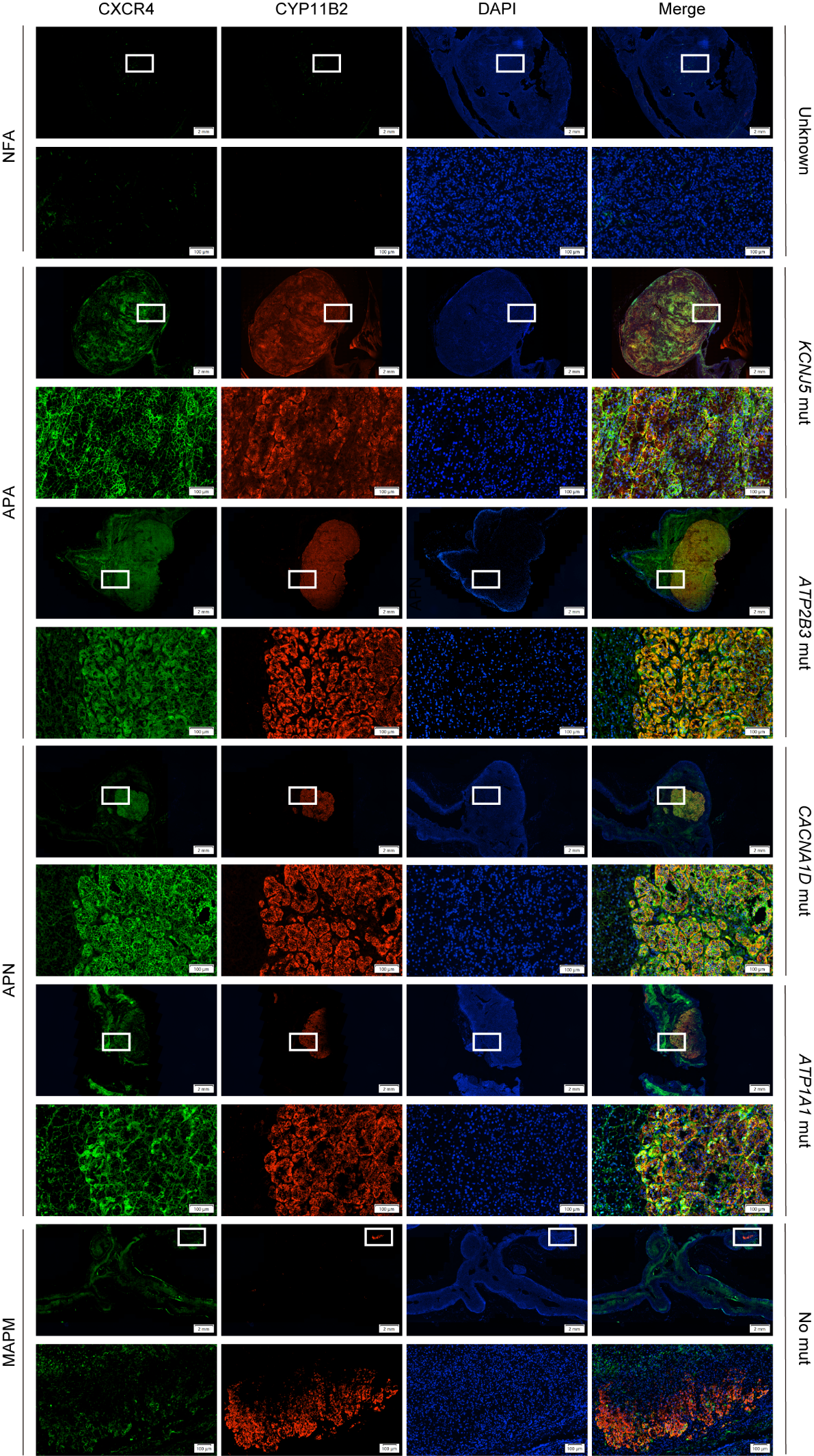
 Figure S2. CXCR4 and CYP11B2 immunofluorescent staining in the adrenal samples of NFA (n=1) and UPA (n=12). Scale bars, 2 mm, 100 μm.**

**
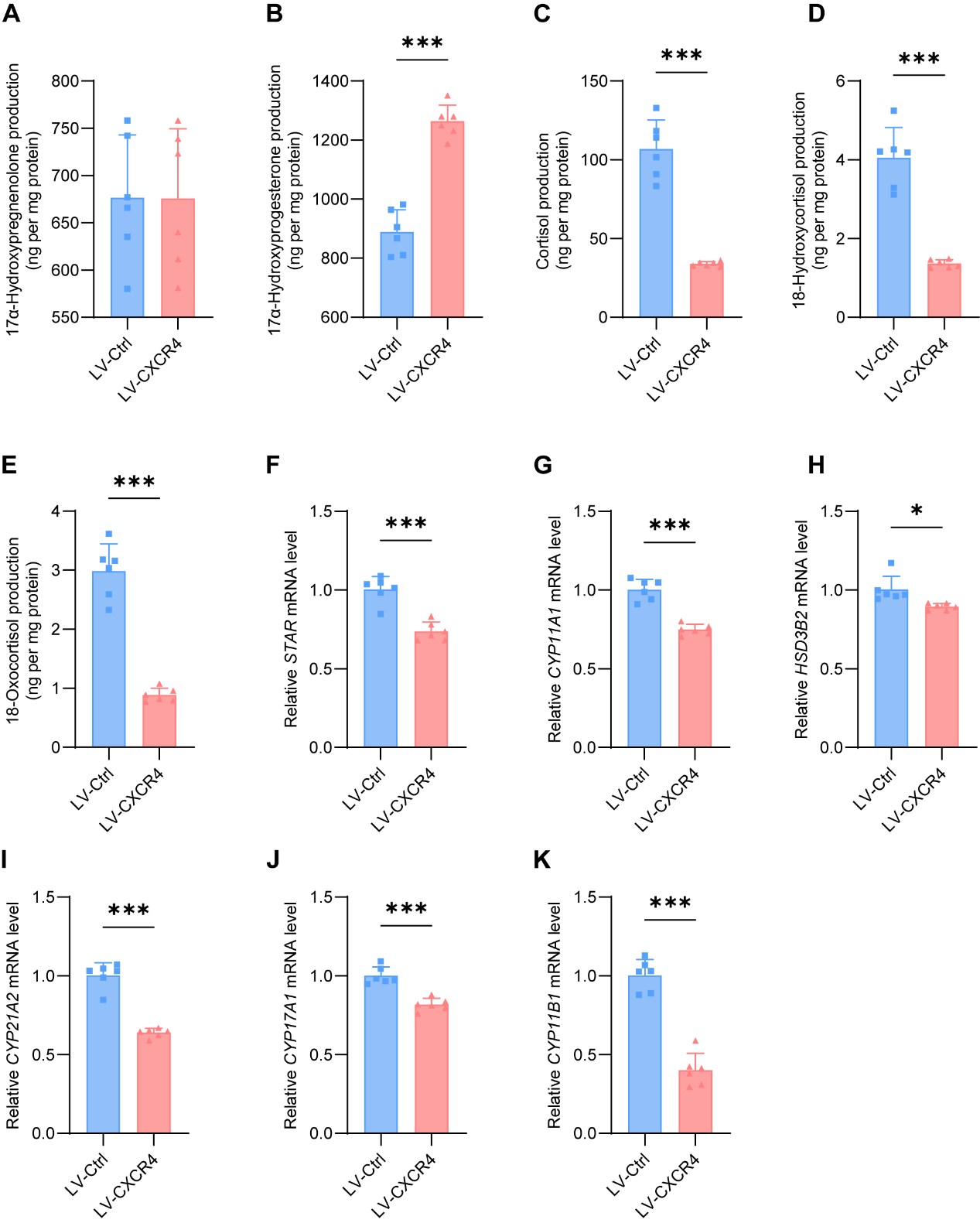
**

**Figure S3. Steroid hormone production and expression of steroidogenic enzymes in H295R Cells following CXCR4 overexpression.**

A-E, Steroid hormone levels (ng per mg protein) in the supernatant of H295R cells after CXCR4 overexpression, including 17α-Hydroxypregnenolone (A), 17α-Hydroxyprogesterone (B), Cortisol (C), 18-Hydroxycortisol (D), and 18-Oxocortisol (E) (n=6). F-K, mRNA levels of steroidogenic enzymes in H295R cells following CXCR4 overexpression, including *STAR* (F), *CYP11A1* (G), *HSD3B2* (H), *CYP21A2* (I), *CYP17A1* (J), and *CYP11B1* (K) (n=6). Data are presented as mean ± SD. **P<0.05*, ***P<0.01*, and ****P<0.001*.

**
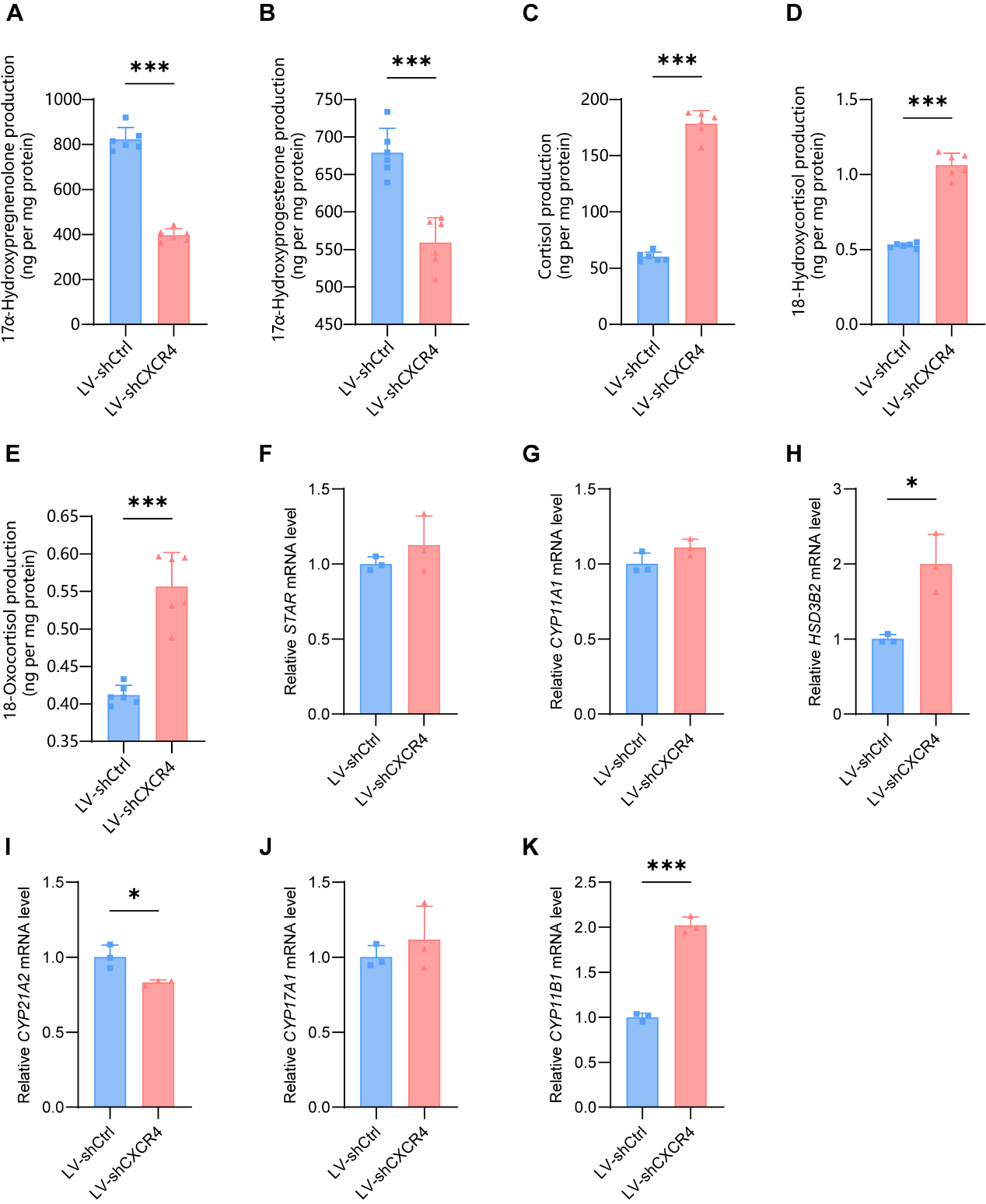
**

**Figure S4. Steroid hormone production and expression of steroidogenic enzymes in H295R Cells following CXCR4 knockdown.**

A-E, Steroid hormone levels (ng per mg protein) in the supernatant of H295R cells after CXCR4 knockdown, including 17α-Hydroxypregnenolone (A), 17α-Hydroxyprogesterone (B), Cortisol (C), 18-Hydroxycortisol (D), and 18-Oxocortisol (E) (n=6). F-K, mRNA levels of steroidogenic enzymes in H295R cells following CXCR4 knockdown, including *STAR* (F), *CYP11A1* (G), *HSD3B2* (H), *CYP21A2* (I), *CYP17A1* (J), and *CYP11B1* (K) (n=3). Data are presented as mean ± SD. **P<0.05*, ***P<0.01*, and ****P<0.001*.

**
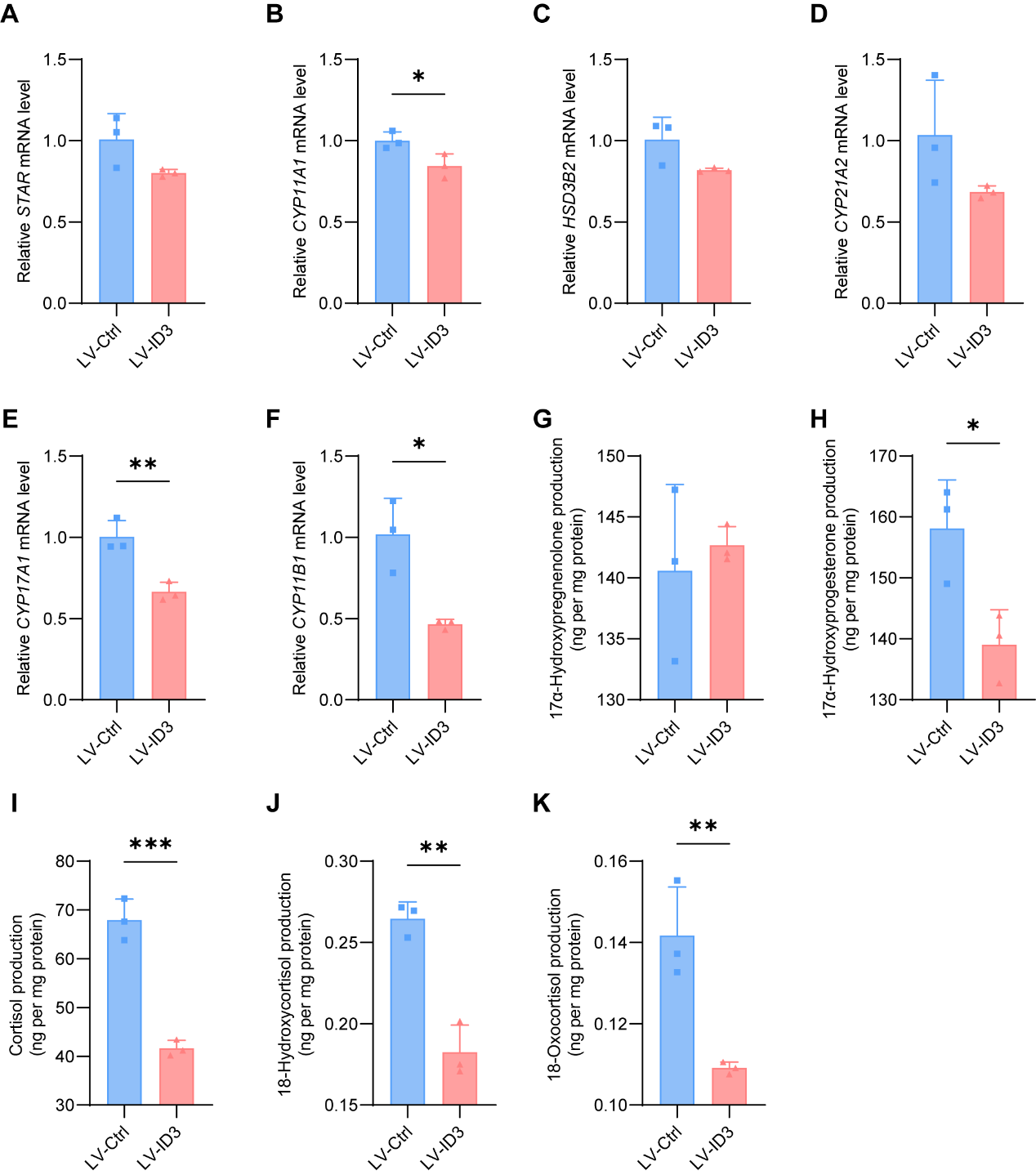
**

**Figure S5. Steroid hormone production and expression of steroidogenic enzymes in H295R Cells following** **ID3 overexpression.**

A-F, mRNA levels of steroidogenic enzymes in H295R cells following ID3 overexpression, including *STAR* (A), *CYP11A1* (B), *HSD3B2* (C), *CYP21A2* (D), *CYP17A1* (E), and *CYP11B1* (F) (n=3). G-K, Steroid hormone levels (ng per mg protein) in the supernatant of H295R cells after ID3 overexpression, including 17α-Hydroxypregnenolone (G), 17α-Hydroxyprogesterone (H), Cortisol (I), 18-Hydroxycortisol (J), and 18-Oxocortisol (K) (n=3). Data are presented as mean ± SD. **P<0.05*, ***P<0.01*, and ****P<0.001*.

**
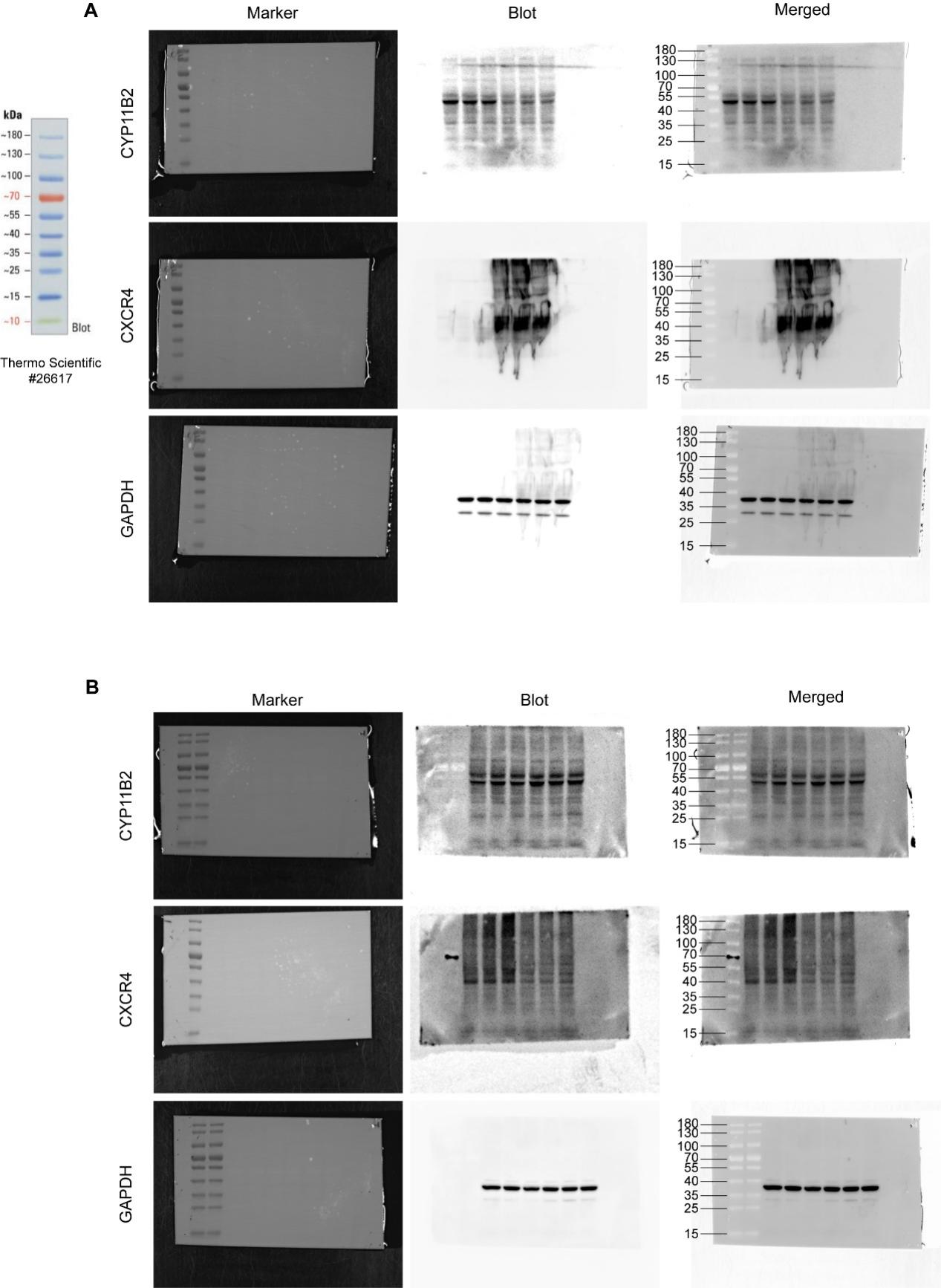
**

**Figure S6. A, B, The entire Western Blot of CYP11B2 and CXCR4 in H295R cells with CXCR4 overexpression (A) or knockdown (B).**

**
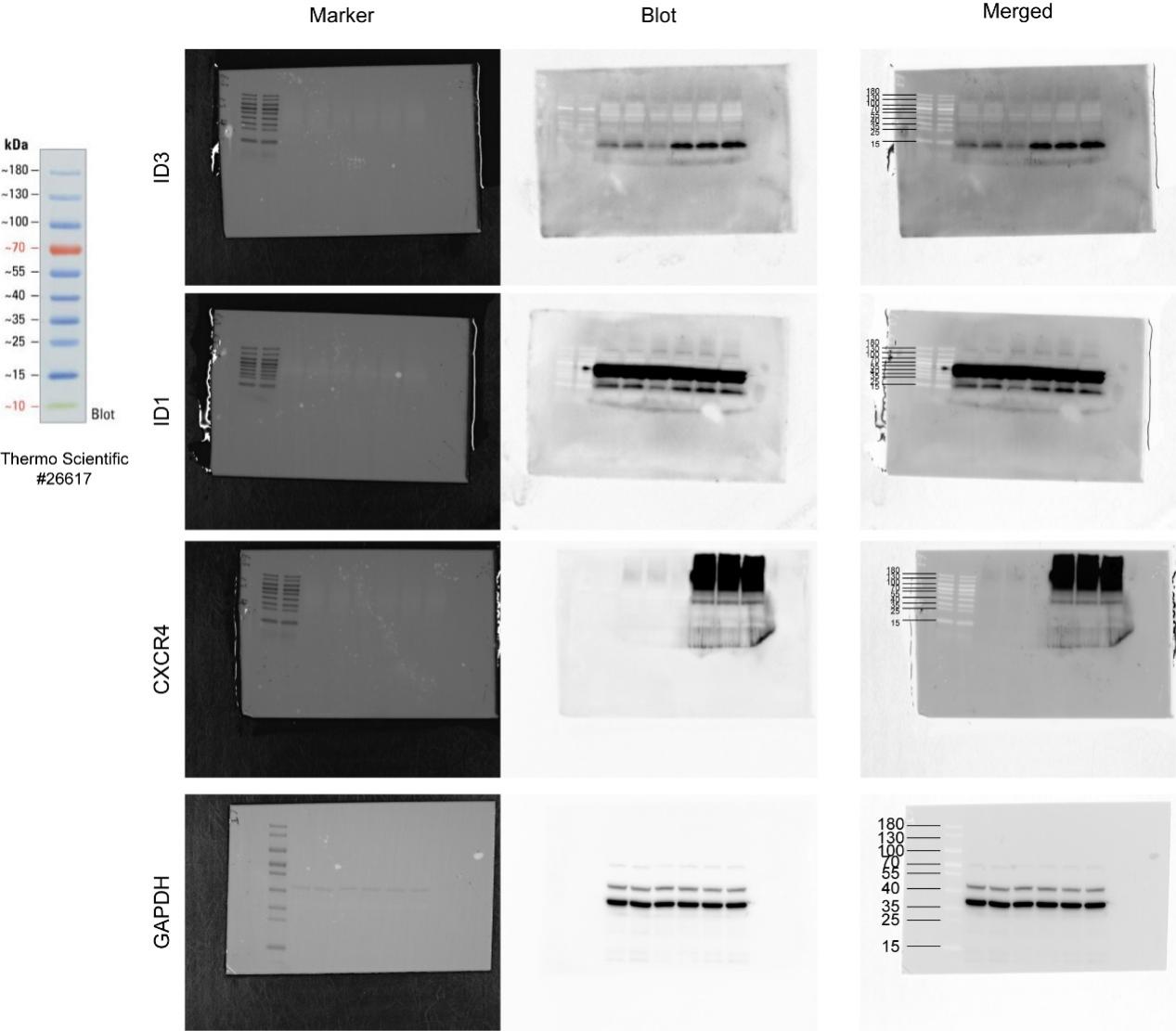
**

**Figure S7. The entire Western Blot of ID3, ID1 and CXCR4 in H295R cells with CXCR4 overexpression.**

**
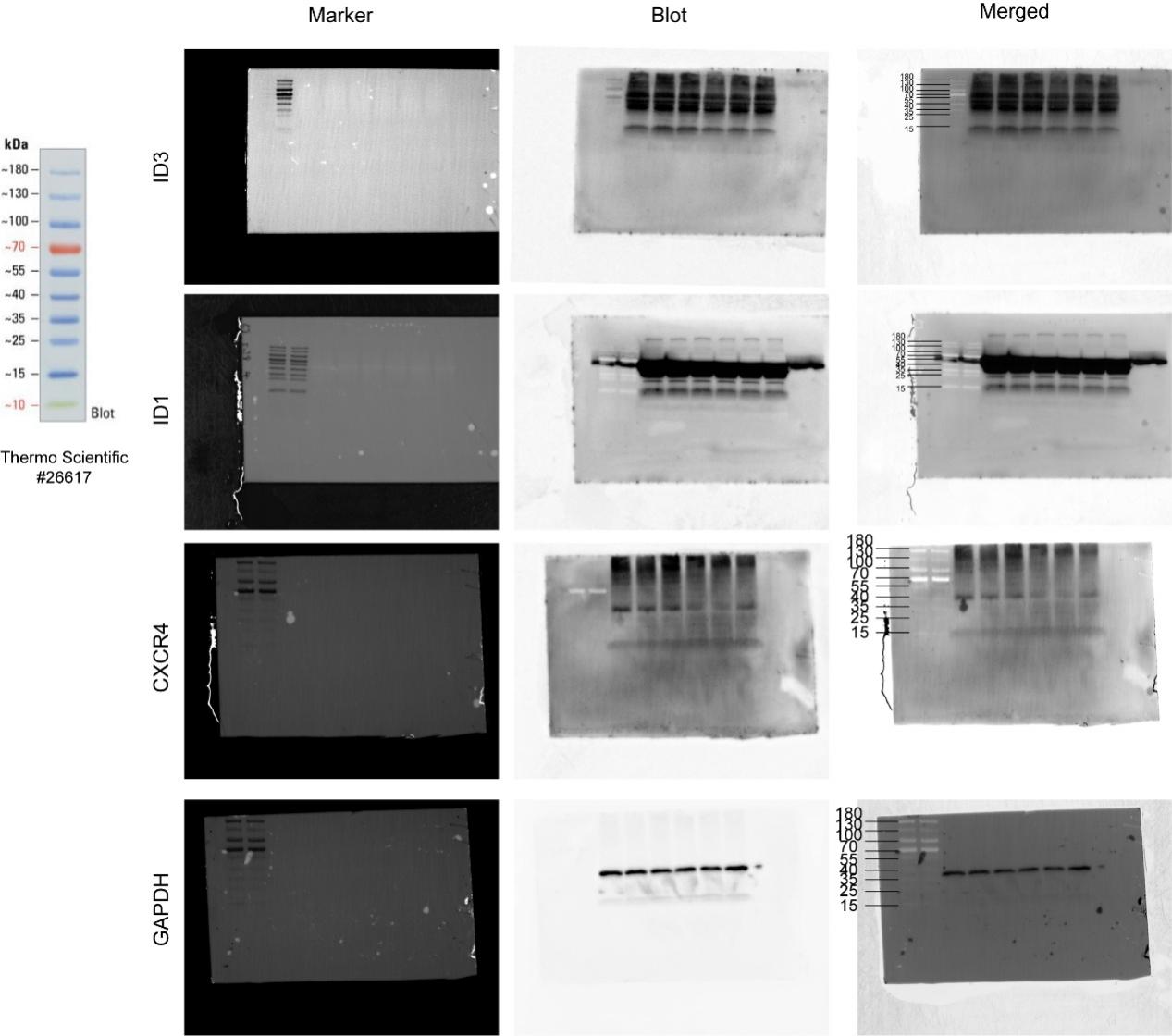
**

**Figure S8. A, The entire Western Blot of ID3, ID1 and CXCR4 in H295R cells with CXCR4 knockdown.**

**
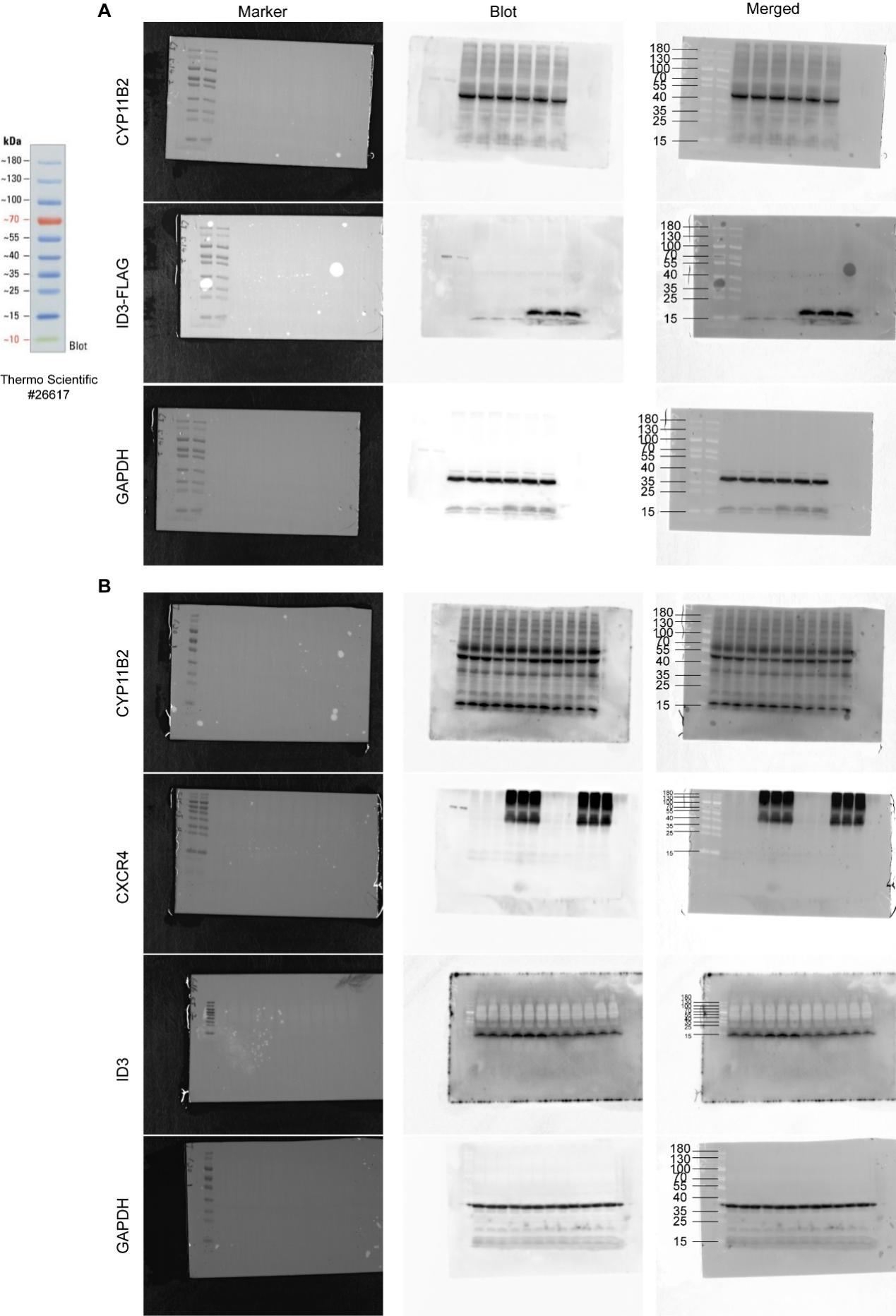
**

**Figure S9. A, The entire Western Blot of CYP11B2 and ID3 in H295R cells with ID3 overexpression. B, The entire Western Blot of CYP11B2, CXCR4 and ID3 in H295R cells overexpressing CXCR4 with or without ID3 knockdown.**
